# Atypical Takotsubo Cardiomyopathy Secondary to Combination of Chemo-Immunotherapy in a Patient With Non-Small Cell Lung Cancer

**DOI:** 10.7759/cureus.9429

**Published:** 2020-07-27

**Authors:** Noman Ahmed Jang Khan, Toni Pacioles, Mohamed Alsharedi

**Affiliations:** 1 Hematology and Oncology, Joan C. Edwards School of Medicine at Marshall University, Huntington, USA

**Keywords:** takotsubo cardiomyopathy, cancer immunotherapy, chemotherapy-related toxicity

## Abstract

Takotsubo cardiomyopathy (TC) also known as broken heart syndrome or stress-induced cardiomyopathy is a relatively rare and transient form of cardiomyopathy. It usually mimics myocardial infarction in terms of clinical and electrocardiographic presentation, but coronary angiography usually does not reveal any evidence of coronary artery occlusion. Even though many underlying causes including emotional, physical or physiological stress have been identified, the exact pathogenesis remains uncertain. Few of anticancer therapies have been reported as an emerging cause of TC; however, no strong evidence of immunotherapy causing cardiomyopathy. We here present a very rare case of atypical TC in a 57-year-old female with advanced stage non-small cell lung cancer who underwent combined cytotoxic chemotherapy and immunotherapy with carboplatin, pemetrexed and pembrolizumab.

## Introduction

Takotsubo, a Japanese word which means “Octopus pot”, was used first by Dote and colleagues in 1990 for certain cardiomyopathy characterized by apical ballooning resembling an octopus pot [1]. The diagnosis of Takotsubo cardiomyopathy (TC) is made by clinical features mimicking an acute myocardial infarction in the absence of obstructive coronary artery disease. Left ventriculography and echocardiogram usually show apical hypokinesis and basal hyperkinesis [2]. Mayo Clinic diagnostic criteria usually aid in the diagnosis [3]. Several chemotherapeutic agents such as 5-fluorouracil (5 FU), capecitabine, cisplatin/docetaxel, cytarabine and cytarabine/daunorubicin have been identified as a risk factor for TC. Combination of carboplatin and pemetrexed, a favored first line regimen for non-small cell lung cancer for a decade is not yet known to be associated with this entity. Immunotherapy, a revolutionary era of cancer treatment with different horizon of adverse effects and toxicities, however apart from rare myocarditis and only one reported case of TC with Ipilimumab, has not been strongly linked to TC [4].

## Case presentation

**Figure 1:**
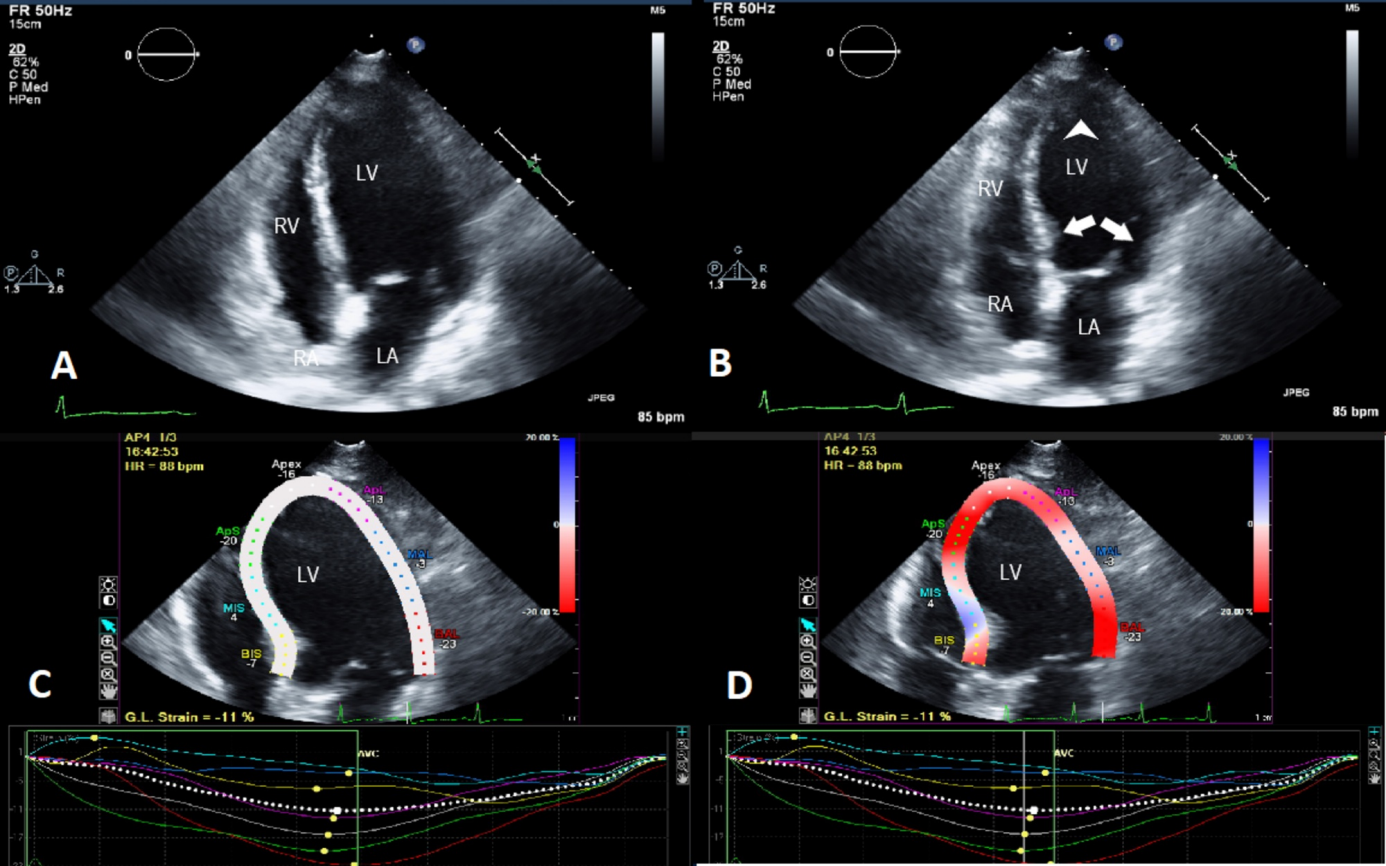
Transthoracic echocardiogram. Panel A represents the four chamber view in end diastole showing global dilatation of left ventricle. Panel B represents four chamber view in end systole demonstrating akinetic left ventricle with sparing of the apical (arrowhead) and basal (arrows) segments. Panel C represents left ventricle wall motion in end diastole and Panel D represents left ventricle wall motion in end systole demonstrating contractility in the basal and apical area with akinesia in the other segments. (LV: Left ventricle, RV: Right ventricle, LA: Left atrium, RA: Right atrium)

**Figure 2:**
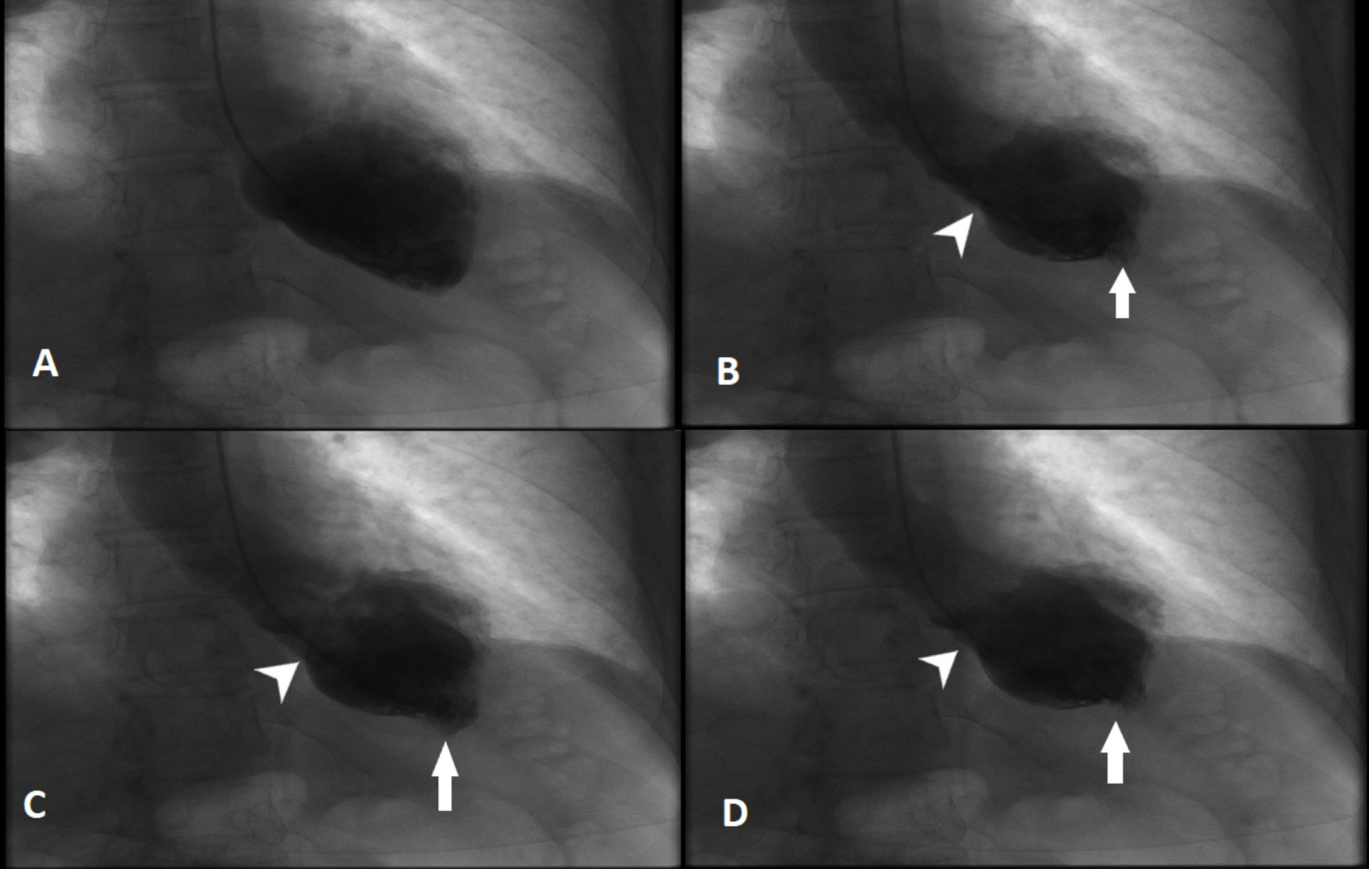
Left ventriculogram. Panel A represents end diastole showing dilatation of the left ventricle. Panel B, C and D represents end systole showing hyperkinetic apical (arrows) and basal segments (arrowheads).

A 57-year-old Caucasian female with no significant cardiac history presented. She was recently diagnosed with advanced stage non-small cell lung cancer, adenocarcinoma. The patient was undergoing systemic therapy consistent of two cytotoxic agents (carboplatin and pemetrexed) combined with anti-PDL-1 check point inhibitor, pembrolizumab on day 1 of every 21 days cycle. She tolerated therapy well with no significant morbidities, however two weeks following cycle number 4, she presented with complaint of fever for five to seven days. On initial clinical evaluation, her vital signs included temperature 102.3 F, pulse rate elevated at 120 beats per minute, blood pressure 115/70 mm Hg. Laboratory workup was unremarkable except for hemoglobin and platelets low at 8.3 g/dl and 59,000, respectively. Computed tomography of chest was performed which came back remarkable for right upper lobe pneumonia obscuring the previously seen right upper lobe neoplasm. The patient was resuscitated with intravenous fluids, broad spectrum antibiotics were initiated, cultures were sent, and the patient was admitted for further medical management. On the 2nd hospital day, the patient noticed worsening chest pain, palpitations, tachypnea and tachycardia. Emergent chest X-ray, electrocardiogram (EKG) and troponins were obtained. EKG revealed sinus tachycardia with no acute ST-T wave changes, but the troponins came back remarkably elevated at 7.5 ng/ml. The patient was started on heparin infusion and cardiac catheterization was planned. Transthoracic echocardiogram was performed in the meantime which showed ejection fraction (EF) 40-45% with severe mid inferior septum, mid inferolateral, mid anterolateral, mid anterior septum, mid inferior and mid anterior wall hypokinesia, sparing the apical and basal segments consistent with atypical Takotsubo cardiomyopathy (TC) (Figure 1). Cardiac catheterization revealed 60% occlusion of proximal right circumflex artery (RCA), abnormal wall motion in the mid-ventricular area with sparing of basal and apical segments consistent with atypical TC (Figure 2). Guideline-directed heart failure treatment was initiated and the patient was discharged in a stable condition. Apart from the recently administered combination chemo-immunotherapy, the patient did not report any recent emotional or stressful events nor did she have any clinical features of pheochromocytoma. This is a very rare case of Takotsubo cardiomyopathy associated with chemo-immunotherapy and to our knowledge this is first case report in medical literature of TC associated with combined chemo-immunotherapy with combination carboplatin, pemetrexed and pembrolizumab.

## Discussion

Takotsubo cardiomyopathy (TC), also known as broken heart syndrome, apical ballooning syndrome or stress cardiomyopathy, is a rare but transient form of left ventricular systolic dysfunction. It accounts for approximately 1 to 2 percent of patients presenting with troponin-positive suspected acute coronary syndrome (ACS) or suspected ST-elevation myocardial infarction [2]. It mimics ACS but in the absence of clinically significant obstructive coronary artery disease. It predominantly affects postmenopausal women and older adults with most of the cases being attributed to some sort of physiological or emotional stress [5].

The exact pathogenesis of TC is unclear. Some of the proposed mechanisms include microvascular coronary artery dysfunction, coronary artery vasospasm, estrogen deficiency and catecholamine excess. The catecholamine excess-induced direct myocardial toxicity and microvascular dysfunction are the most favored ones [6]. Acute substernal chest pain is the most common presenting symptom whereas other common symptoms include dyspnea and tachyarrhythmias and syncope [5]. Cardiac biomarkers are elevated in majority of cases but in the absence of clinically significant coronary artery occlusion. Very few patients will present with cardiogenic shock and left ventricular circulatory dysfunction.

Recently, many chemotherapeutic agents have been linked to TC including 5-fluorouracil (5 FU), capecitabine, cisplatin/docetaxel, cytarabine, cytarabine/daunorubicin [7]. The pathogenesis of chemotherapy-induced TC remains uncertain but is mostly attributed to the emotional and physiological stress related to underlying malignancy, direct cardiotoxicity caused by the free radicals and possible paraneoplastic phenomenon [8]. Our patient received four cycles of carboplatin (a platinum-based antineoplastic agent), pemetrexed (a folic acid metabolism inhibitor) and pembrolizumab (a programmed death-1 monoclonal antibody). In literature, none of these agents have been found to be associated with any cardiovascular complications including TC except for some cases of myocarditis and pericarditis noticed with pembrolizumab [9, 10].

Treatment of TC is mainly supportive including identifying the underlying emotional and physiological stressors. Heart failure in TC is managed according to the guidelines with extra care advised to patients with left ventricular outflow tract obstruction as preload reduction can have detrimental impacts in such cases [11]. Chemotherapy agents should be discontinued and the decision to rechallenge patients with similar agents after the recovery of cardiac function should be strongly individualized as no clear guidelines have been established yet [12].

Cardiac function will recover in most patients, but the in-hospital mortality rates remain high (2 to 8 %) [13]. In one study, in-hospital mortality with TC was 8.9%, a nonfatal recurrence rate of 1.8% and mean survival of 4.47 years (95% confidence interval 3.81 to 5.13). QTc interval on presentation with TC was predictive of outcome in the similar study [10]. Several risk factors including age > 70, left ventricular systolic function < 40%, presence of other comorbidities and underlying stressors are associated with poor outcomes [14, 15].

## Conclusions

Even though underlying malignancy puts patients under enough physiological and emotional stress to cause stress-induced cardiac dysfunction, theoretically, the administration of systemic cancer therapy may enhance this risk; however, neither pemetrexed with carboplatin are not known to induce TC nor single agent pembrolizumab. As a first reported case, this case highlights the importance of possible causation between Takotsubo cardiomyopathy and new horizon of systemic therapy with combination of cytotoxic agent and immunotherapy. Physician awareness of this potential entity, prompt identification and early intervention are important. Further studies are warranted to investigate the pathophysiology of TC and its association with chemo-immunotherapy.
